# Supplementary figures and images for: Personality variation is eroded by simple social behaviours in collective foragers
Source: PLoS Comput Biol. 2023 Mar 2;19(3):e1010908. doi: 10.1371/journal.pcbi.1010908 (PMC9980820; doi:10.1371/journal.pcbi.1010908)

central

nearest neighbour

majority

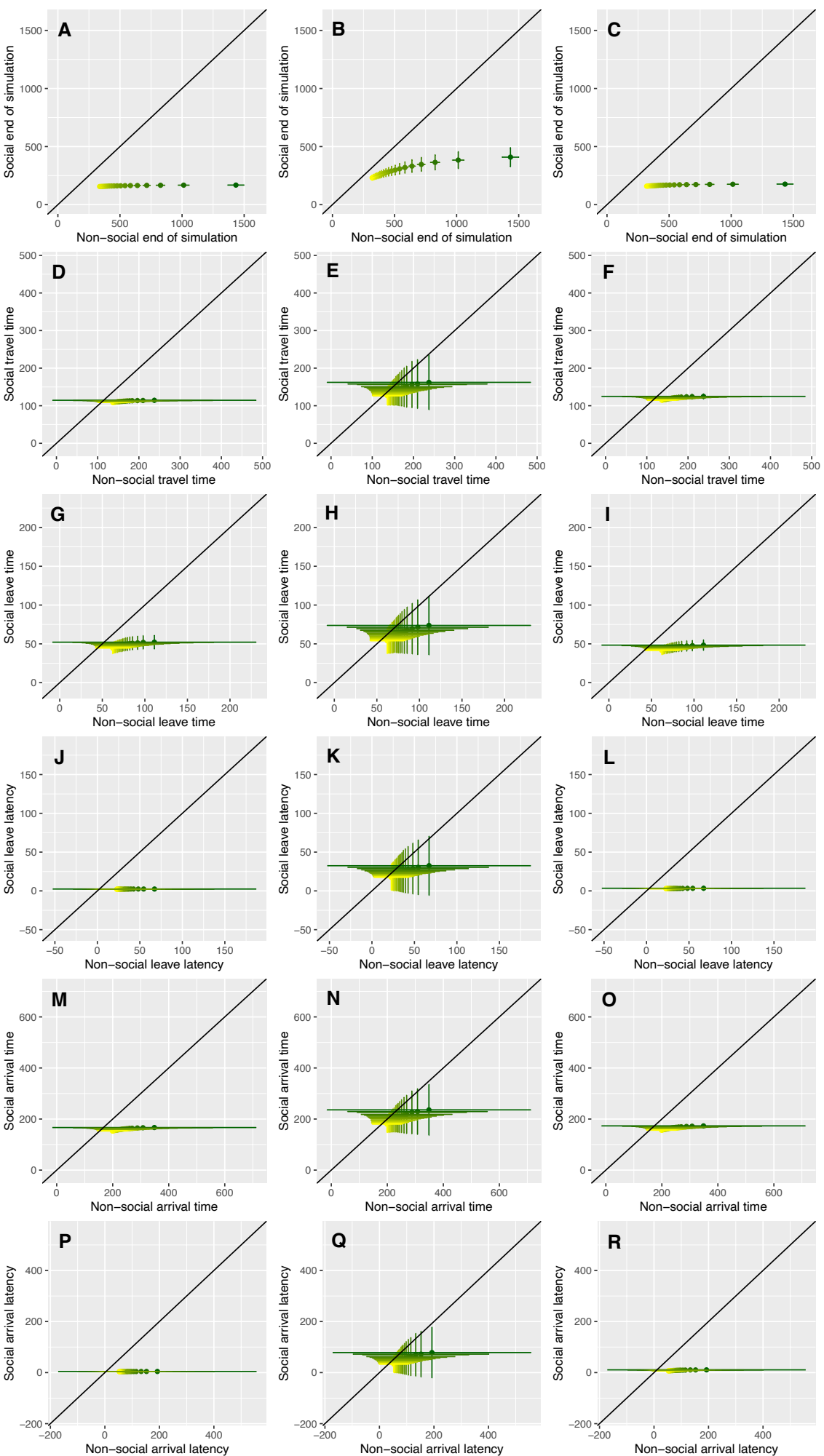

Supplement: S1 Fig — Within each panel, pbaseline is systematically altered increased from 0.0001 (dark green) to 0.0020 (yellow), with the colour gradient representing equally-sized increments of 0.0001 between these values, and other parameters are set as described in the ’model exploration’ methods. See legend for Fig 3 for more detail. (PDF) [file pcbi.1010908.s001.pdf]

central

nearest neighbour

majority

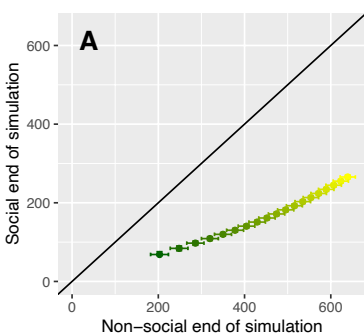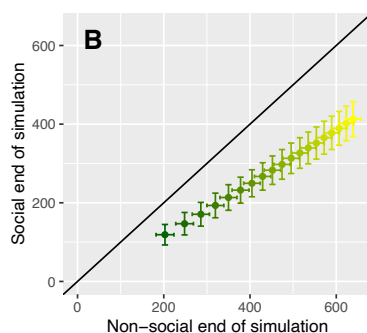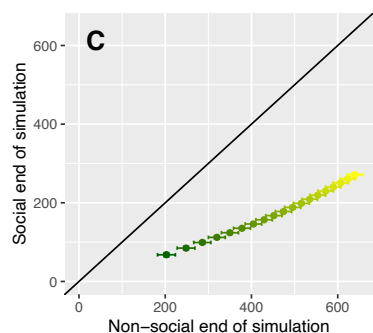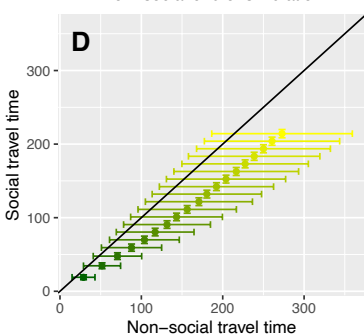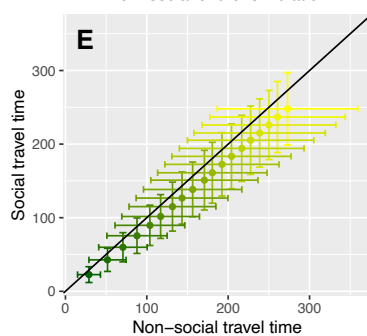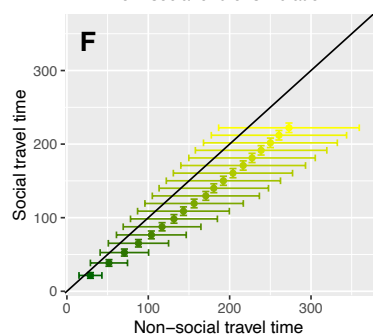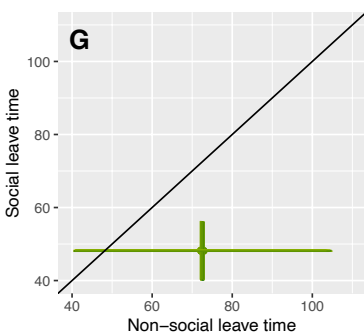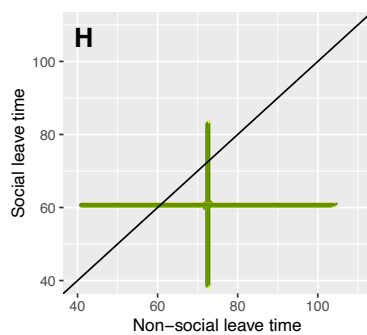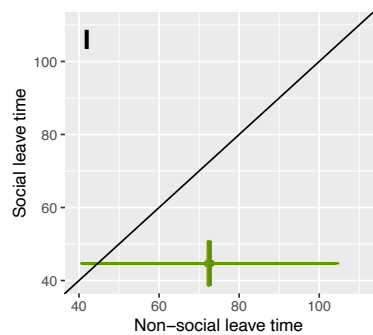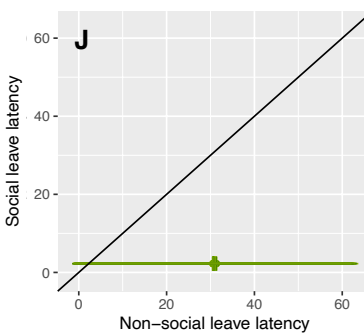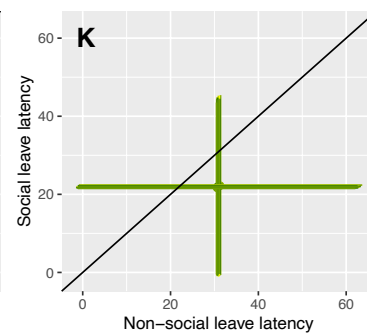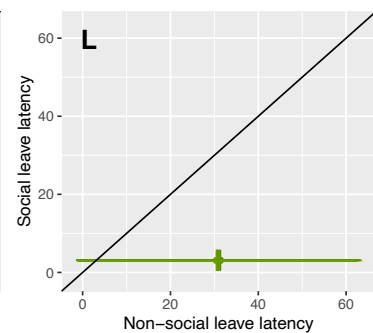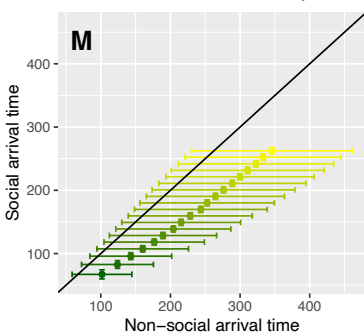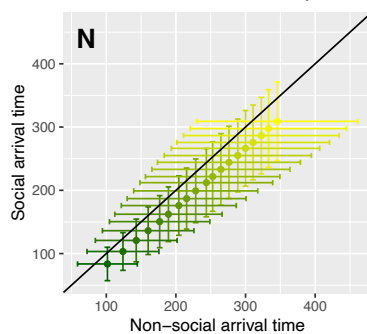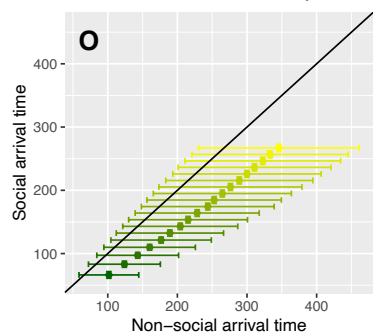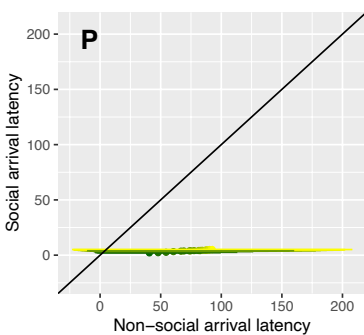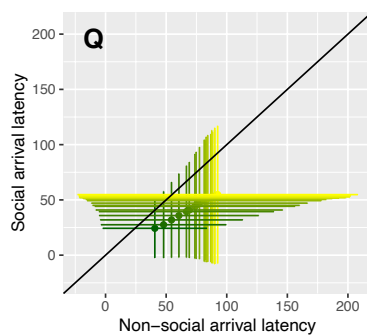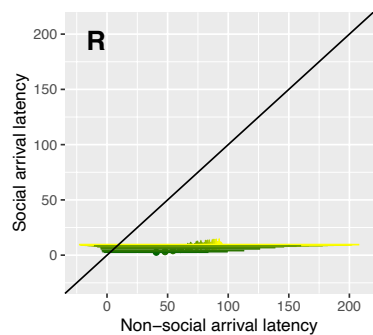

Supplement: S2 Fig — Within each panel, dfood is systematically altered between 20 (dark green) and 200 distance units (yellow), with the colour gradient representing equally-sized increments of 10 units between these values, and other parameters are set as described in the ’model exploration’ methods. See legend for Fig 3 for more detail. (PDF) [file pcbi.1010908.s002.pdf]

central

nearest neighbour

majority

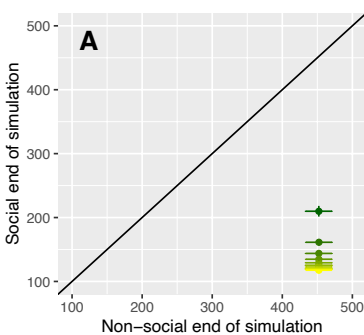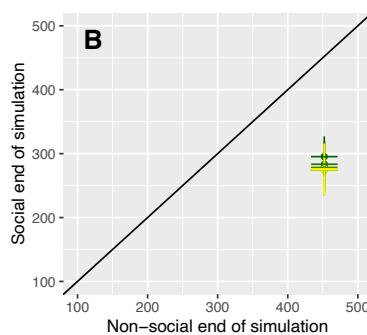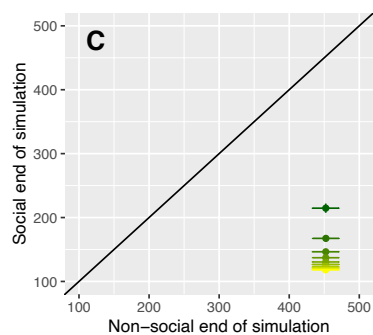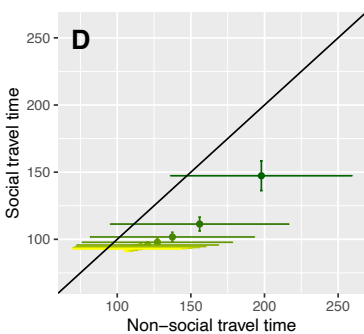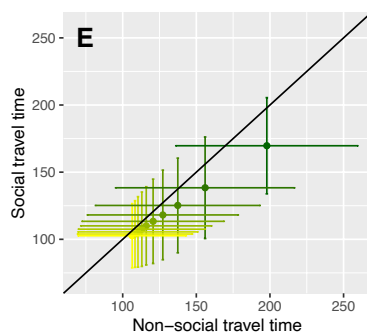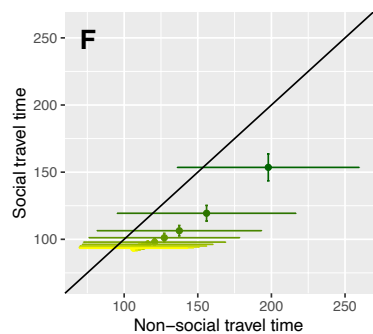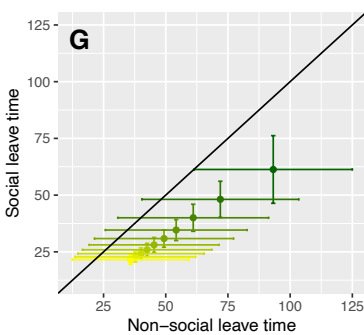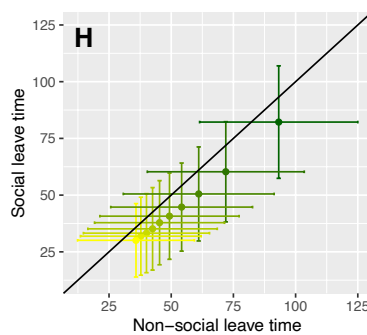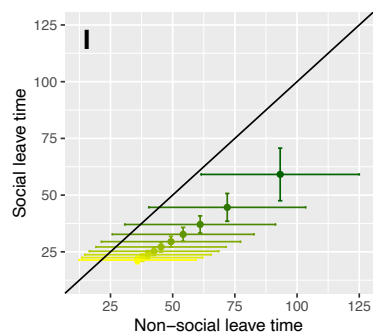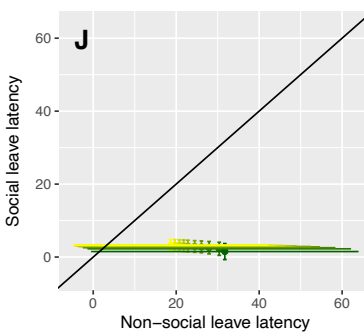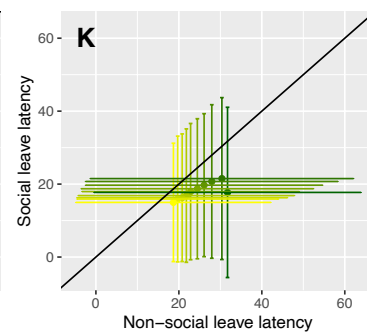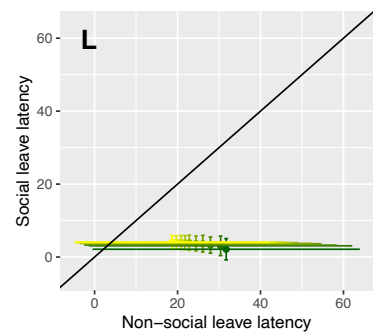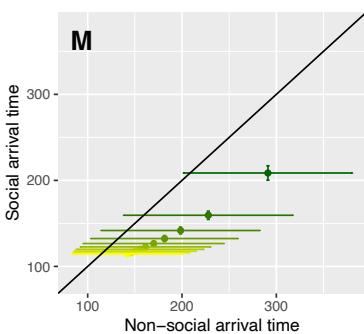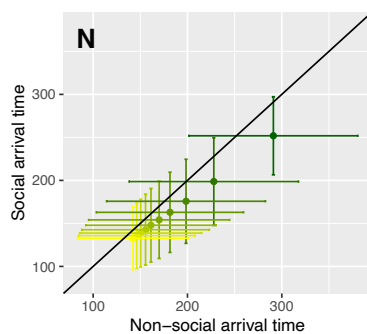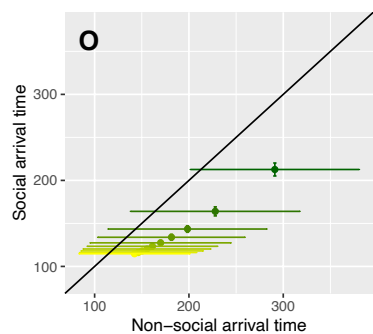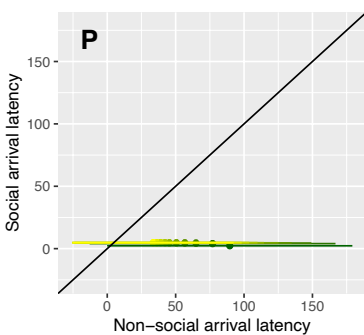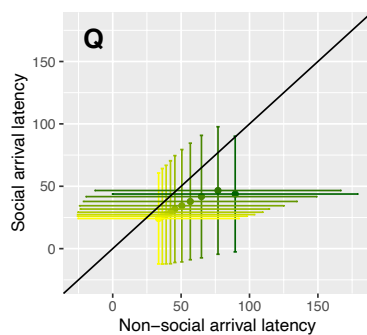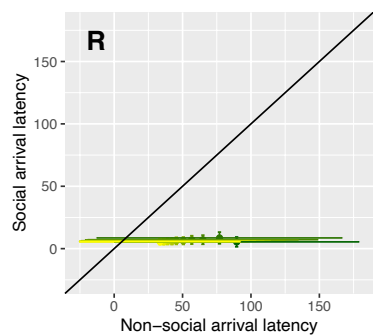

Supplement: S3 Fig — Within each panel, n is systematically altered between 5 (dark green) and 50 individuals (yellow), with the colour gradient representing equally-sized increments of 5 individuals between these values, and other parameters are set as described in the ’model exploration’ methods. See legend for Fig 3 for more detail. (PDF) [file pcbi.1010908.s003.pdf]

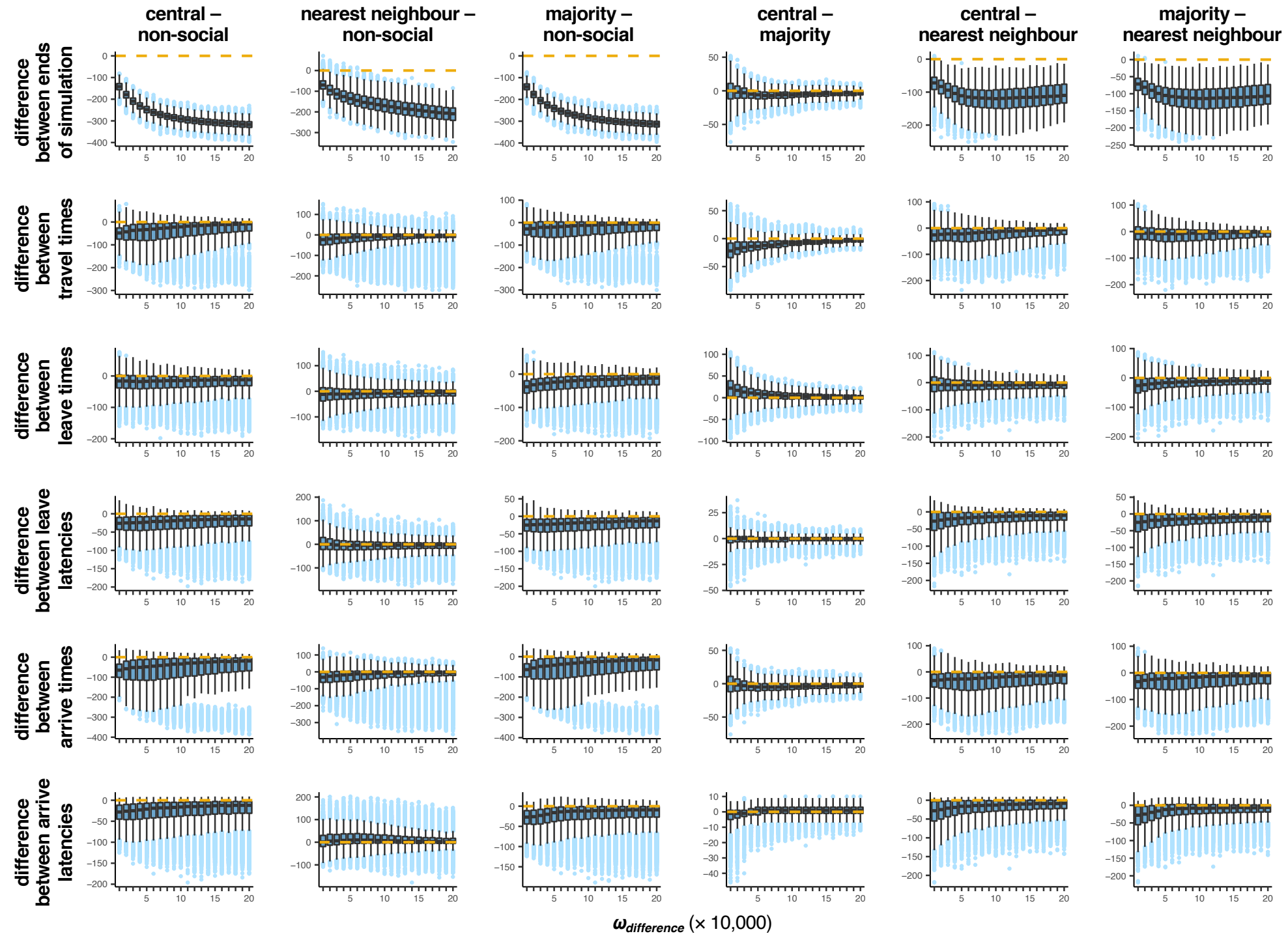

Supplement: S4 Fig — The panels show summaries of the difference in the behavioural metrics (the labels at the left of the figure describe each statistic) when comparing the six possible pairs of social behaviour (indicated by the labels at the top of the figure), noting that the differences were calculated by comparing the behavioural metrics for simulations of groups experiencing the same starting conditions but following different behavioural rules. Boxplots represent the median and quartile values for each simulation, with whiskers representing the minimum of the maximum value and 1.5 × interquartile range, with points representing outliers beyond this range. (PDF) [file pcbi.1010908.s004.pdf]

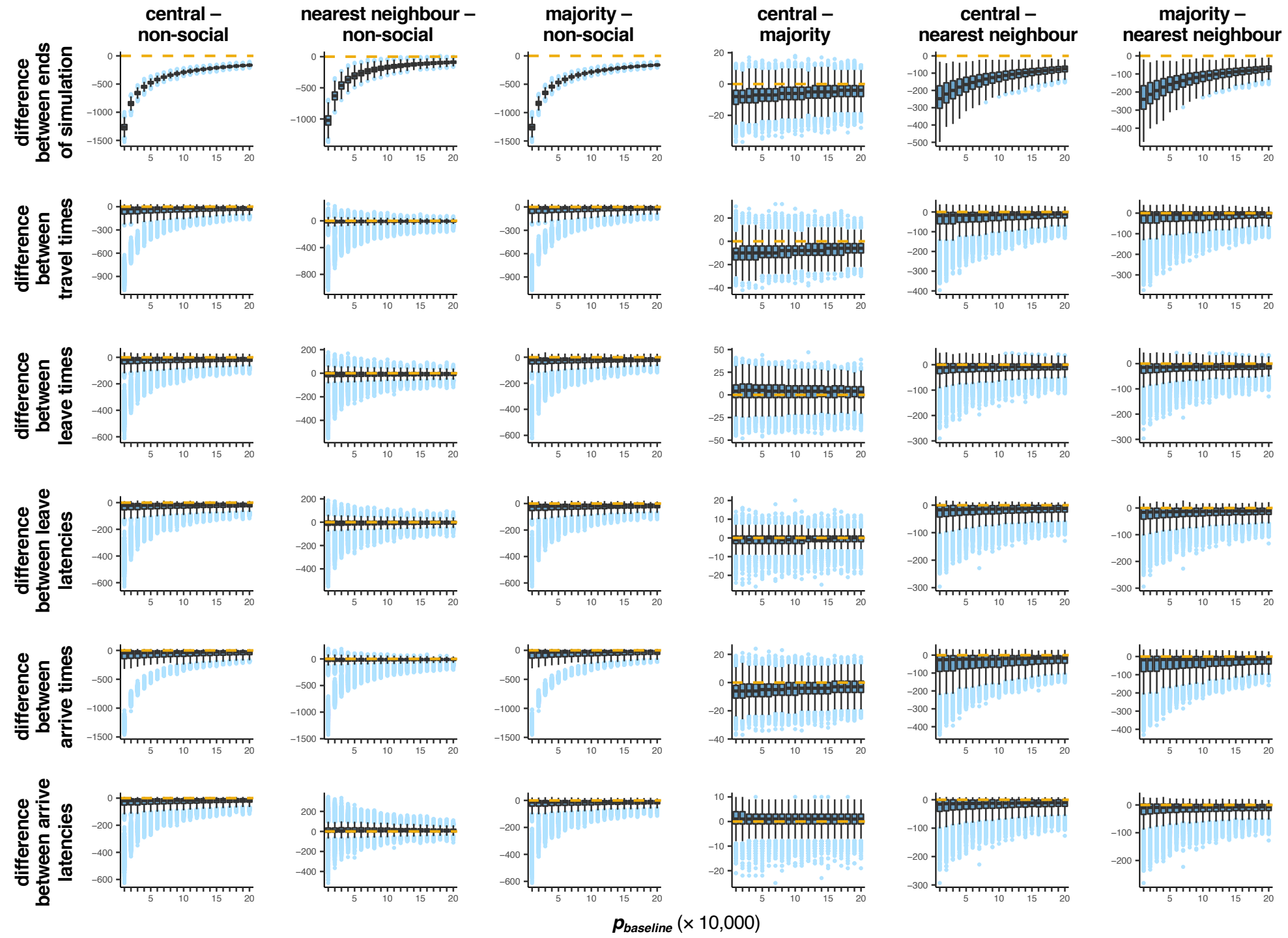

Supplement: S5 Fig — See the legend to S4 Fig for details. (PDF) [file pcbi.1010908.s005.pdf]

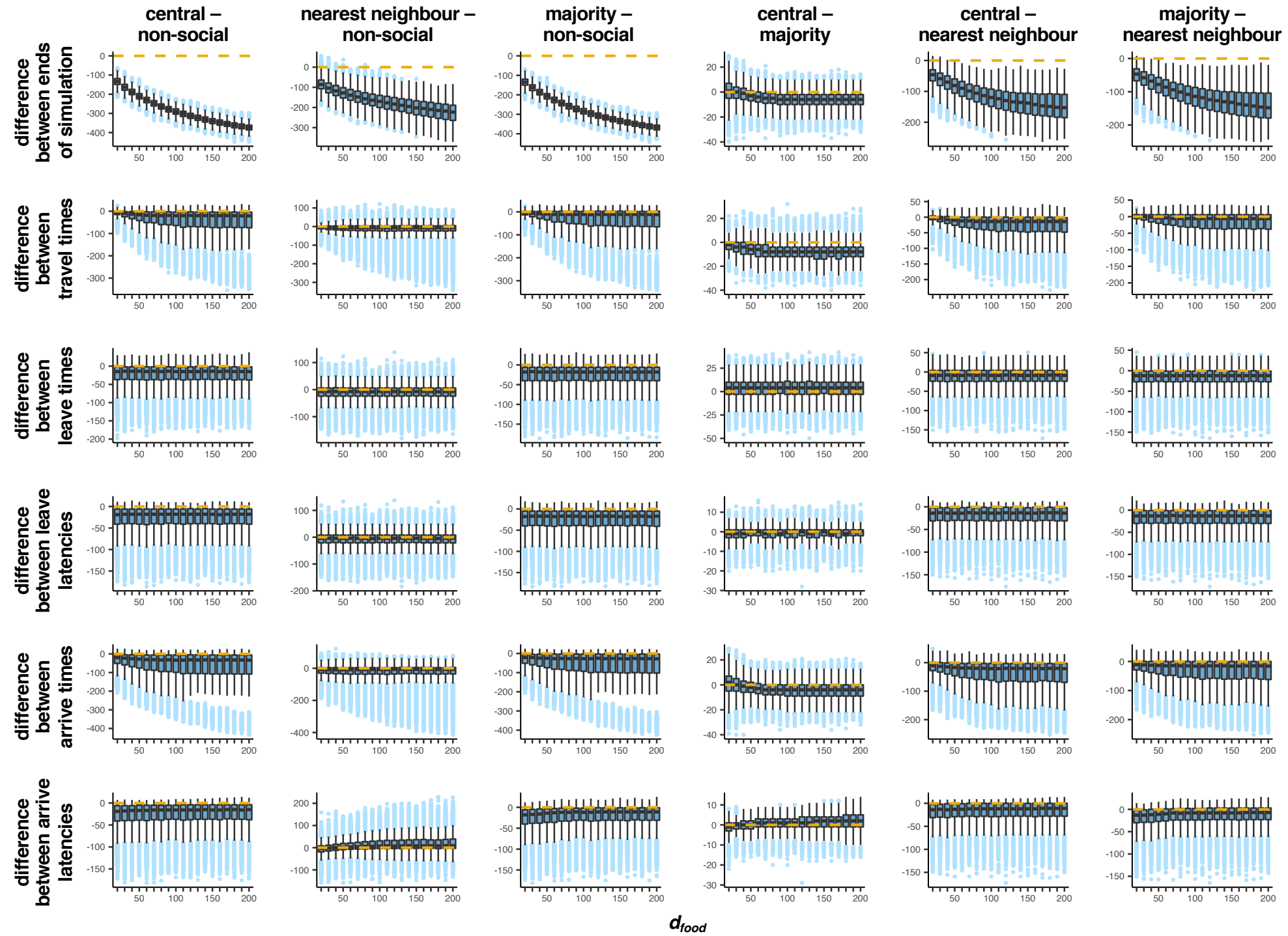

Supplement: S6 Fig — See the legend to S4 Fig for details. (PDF) [file pcbi.1010908.s006.pdf]

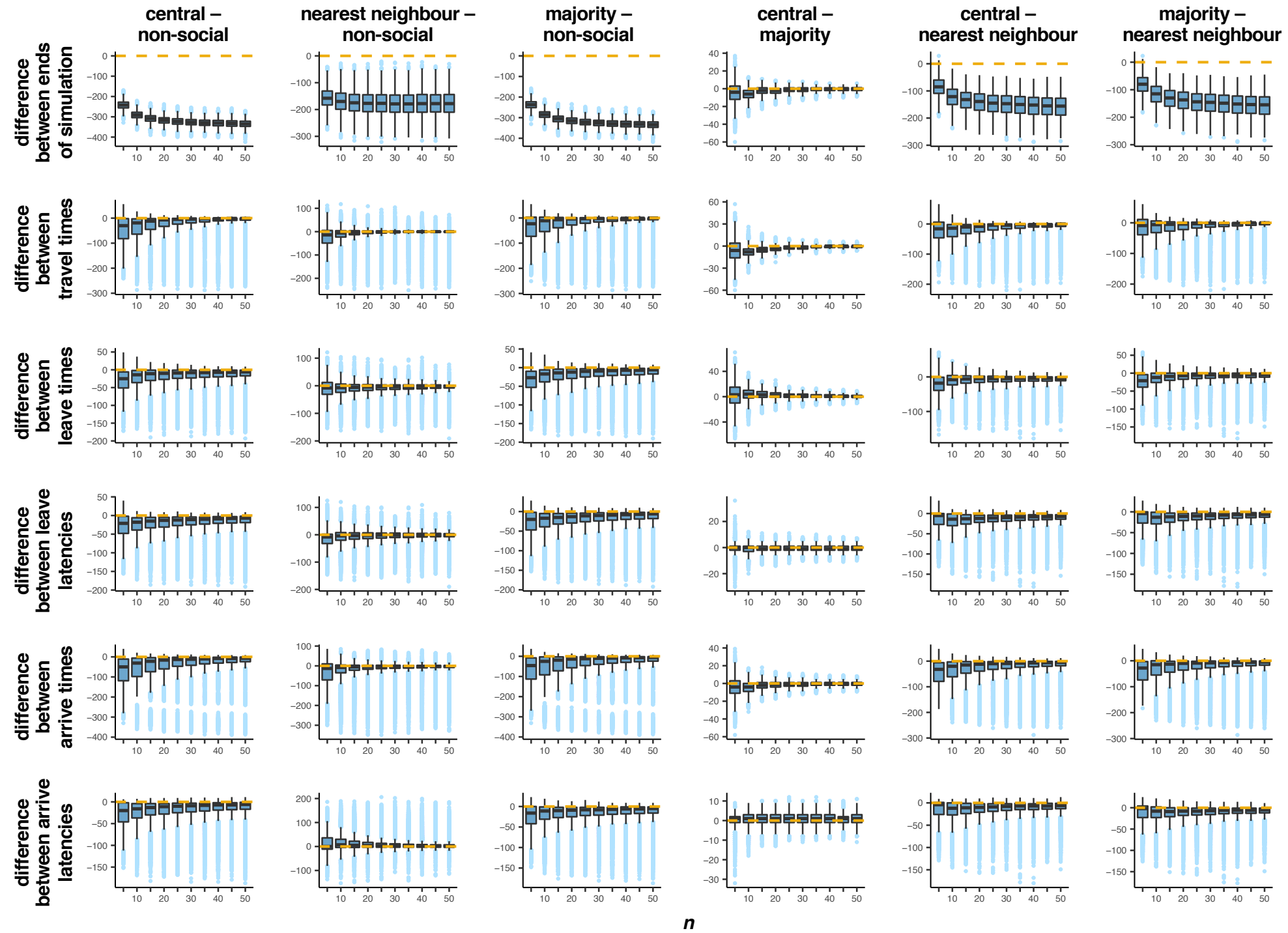

Supplement: S7 Fig — See the legend to S4 Fig for details. (PDF) [file pcbi.1010908.s007.pdf]
